# Novel molecular assays for monitoring T cell function after HSCT in two siblings with variants in *DCLRE1C*

**DOI:** 10.70962/jhi.20250171

**Published:** 2026-02-20

**Authors:** Anna Lyytikäinen, Yasaman Shamshirgaran, Hedvig Hjerpe, Sara Cajander, Emilie Wahren Borgström, Shaghayegh Gharaghani, Elina Erikson, Sofie Vonlanthen, Per Marits, Ola Hammarsten, C.I. Edvard Smith, Anders Fasth, Pegah Johansson

**Affiliations:** 1 Department of Clinical Chemistry, Sahlgrenska University Hospital, Gothenburg, Region Västra Götaland, Sweden; 2 Department of Laboratory Medicine, Sahlgrenska Academy at University of Gothenburg, Gothenburg, Sweden; 3 Department of Infectious Diseases, Faculty of Medicine and Health, Örebro University Hospital, Örebro, Sweden; 4 Department of Infectious Diseases, Karolinska University Hospital, Huddinge, Sweden; 5 Department of Laboratory Medicine, Clinical Immunology, Karolinska Institute, Stockholm, Sweden; 6 Clinical Immunology and Transfusion Medicine, Karolinska University Hospital, Huddinge, Sweden; 7 Department of Laboratory Medicine, Biomolecular and Cellular Medicine, Karolinska Institute, Stockholm, Sweden; 8 Translational Research Center Karolinska, Karolinska University Hospital, Huddinge, Sweden; 9 Department of Pediatrics, Institute of Clinical Sciences, Sahlgrenska Academy at University of Gothenburg, Gothenburg, Sweden

## Abstract

A subgroup of patients with severe combined immunodeficiencies (SCID) has disease-causing variants in genes involved in nonhomologous end-joining (NHEJ) repair of DNA double-strand breaks (DSBs). The most common variants among these patients are in the *DCLRE1C* gene coding for the Artemis protein, which makes up 2–3% of all SCID. Patients with Artemis deficiency are radiosensitive (RS) and are referred to as RS-SCID. Hematopoietic stem cell transplantation (HSCT) is the only curative treatment. Due to defects in NHEJ repair, modified protocols are used for HSCT conditioning, resulting in poor engraftment (post-transplantation) in some patients. We describe the clinical and molecular findings in two siblings with a compound heterozygous variant in DCLRE1C. We previously reported functional radiosensitivity in their T cells using a novel cell division assay (CDA). Here, we applied the CDA and the novel DSB repair assay to assess T cell function after HSCT. Posttransplant T cells showed reduced radiosensitivity and a DNA repair efficiency comparable to controls. Donor engraftment was low in myeloid cells but exceeded 95% in lymphocytes. Both patients had low lymphocyte counts after HSCT, but clinical symptoms improved. The assays presented here are useful for investigating the pathology of new variants in the DSB repair pathway and monitoring outcomes after HSCT.

## Teaser

Lyytikäinen et al. report two novel assays to characterize T cell function in two patients with DCLRE1C variants diagnosed with leaky SCID. The cell division assay is used to quantify radiosensitivity in patient T cells, and the DNA double-strand break repair assay is used to measure repair defects.

## Introduction

Severe combined immunodeficiency (SCID) is a rare, heterogeneous group of diseases characterized by severe T cell lymphopenia and profound defects in T and B cell function. Natural killer (NK) cells are also affected in a subgroup of patients (1, 2). Newborn screening–based incidence ranges from 1 in 58,000 to 1 in 61,000 in the USA, and in Sweden, the estimate is 1 in 38,500 (3, 4, 5, 6). In the absence of curative treatment, most patients do not survive their first few years of life. Clinical presentation includes recurrent and life-threatening bacterial, viral, and fungal infections early in life. Clinical features typically include absent T cells and absent or residual B cells, with or without NK cells. Currently, hematopoietic stem cell transplantation (HSCT) is the main mode of curative treatment (7). Early transplantation before infection onset significantly improves survival, which has led to the implementation of newborn screening for SCID in many countries, including the USA and the Nordic region (6, 8). Variants in ∼20 genes have been linked to SCID, most commonly *IL2RG*, *IL7RA*, *ADA*, and *RAG1/2* (9, 10, 11, 12). However, the causative gene remains unknown in about 7.6% of SCID cases identified by newborn screening (12).

A subset of SCID patients carries variants affecting V(D)J recombination, essential for B and T cell development. These patients exhibit a T^−/low^, B^−^, NK^+^ SCID phenotype. SCID in this group is inherited in an autosomal recessive manner. A minority of these patients have disease-causing variants in genes involved in nonhomologous end-joining (NHEJ), a critical mechanism for repairing DNA double-strand breaks (DSBs), which are intermediates in V(D)J recombination (13). NHEJ also repairs spontaneous DSBs caused by endogenous and exogenous DNA damage, essential for genomic stability. Consequently, patients with SCID due to NHEJ pathway variants are sensitive to DNA-damaging agents such as radiation and are at a high risk of developing cancer. These patients are also referred to as radiosensitive SCID (RS-SCID) (7, 14, 15). The most common disease-causing variants in RS-SCID are in *DCLRE1C*, which encodes for the Artemis protein. These cases account for 2–3% of all SCID (9, 14). *DCLRE1C* mutations are overrepresented in certain populations. Affected individuals present with recurrent respiratory tract infections, candidiasis, immune dysregulation, and malignancies during childhood, adolescence, or even adulthood (16, 17, 18). Due to their defect in NHEJ and sensitivity to DNA-damaging treatments, reduced-intensity pretransplant conditioning is required (19). Despite this, outcomes after allogeneic HSCT are generally poor in this subgroup of patients (20, 21).

Hypomorphic variants that retain partial protein function can result in combined immunodeficiency (CID) with milder or atypical presentations, known as “leaky” SCID (22), as observed in the siblings described here. These patients may have later onset of symptoms and are challenging to diagnose. With the integration of next-generation sequencing (NGS) in clinical diagnostics, new variants in RS-SCID–associated genes are being identified, but many of these variants are of uncertain clinical significance. Familial segregation studies and appropriate functional assays are needed to determine the pathogenicity of these variants and provide appropriate treatment options.

We recently reported the cell division assay (CDA) as a surrogate for a cell survival assay to measure cellular sensitivity to DNA-damaging agents in vitro (23), which is feasible to use with T cell cultures from patients. The assay sensitively and specifically detected DNA repair deficiency in T cells from patients with variants in Fanconi anemia genes (*FANCA*, *FANCB*, *FANCC*, *FANCD1, FANCD2, FANCF*), *PRKDC*, and *DCLRE1C,* based on their survival in response to specific treatments (24, 25). We validated this assay using T cells from individuals with known DNA repair defects and in cells from the siblings carrying the hypomorphic *DCLRE1C* variants (23, 25).

In this study, we report another novel assay that directly quantifies the relative NHEJ and homologous recombination (HR) repair in T cells to evaluate the efficiency of these pathways in patient cells. The assay utilizes the reporter-based flow cytometry method described by Seluanov et al. (26) and was optimized for clinical application in patient T cells.

Furthermore, we report the genetic, molecular, and clinical findings in these two siblings with compound heterozygous *DCLRE1C* variants, one of which has only recently been reported in one other patient (27). We previously described the in vitro radiosensitivity in the T cells from these siblings using the CDA (24). Here, we used the CDA and the NHEJ/HR assay to quantify the relative DSB repair in the patients’ T cells after HSCT to evaluate the functional outcome of transplantation in these patients.

## Results

### Clinical and molecular characteristics

The patients presented here are a sister and a brother diagnosed at the ages of 28 and 25 years, respectively.

The sister presented with cough and failure to thrive in early childhood. She was diagnosed with bronchiectasis and tested PCR-positive for *Pneumocystis jirovecii* in sputum at the age of five. IgA and IgM levels were normal, but IgG and IgG2 were slightly decreased. At age seven, she was started on immunoglobulin replacement therapy, with good clinical response throughout childhood. At 22 years of age, she was treated for HPV infection (six variants), but she still developed cervical carcinoma in situ, which was successfully treated with conization.

The brother had a history of recurrent sinus infections in childhood and underwent endoscopic sinus surgery. He was started on immunoglobulin replacement therapy at the age of two. He had several granulomas, including in his facial skin, which were unresponsive to corticosteroids, methotrexate, and TNFα inhibitors. He was later diagnosed with an aggressive B cell lymphoma and received curative treatment with six cycles of dose-dense R-CHOP-14 (rituximab, cyclophosphamide, doxorubicin, vincristine, and prednisone). He achieved complete remission after three cycles.

**Figure S1.**
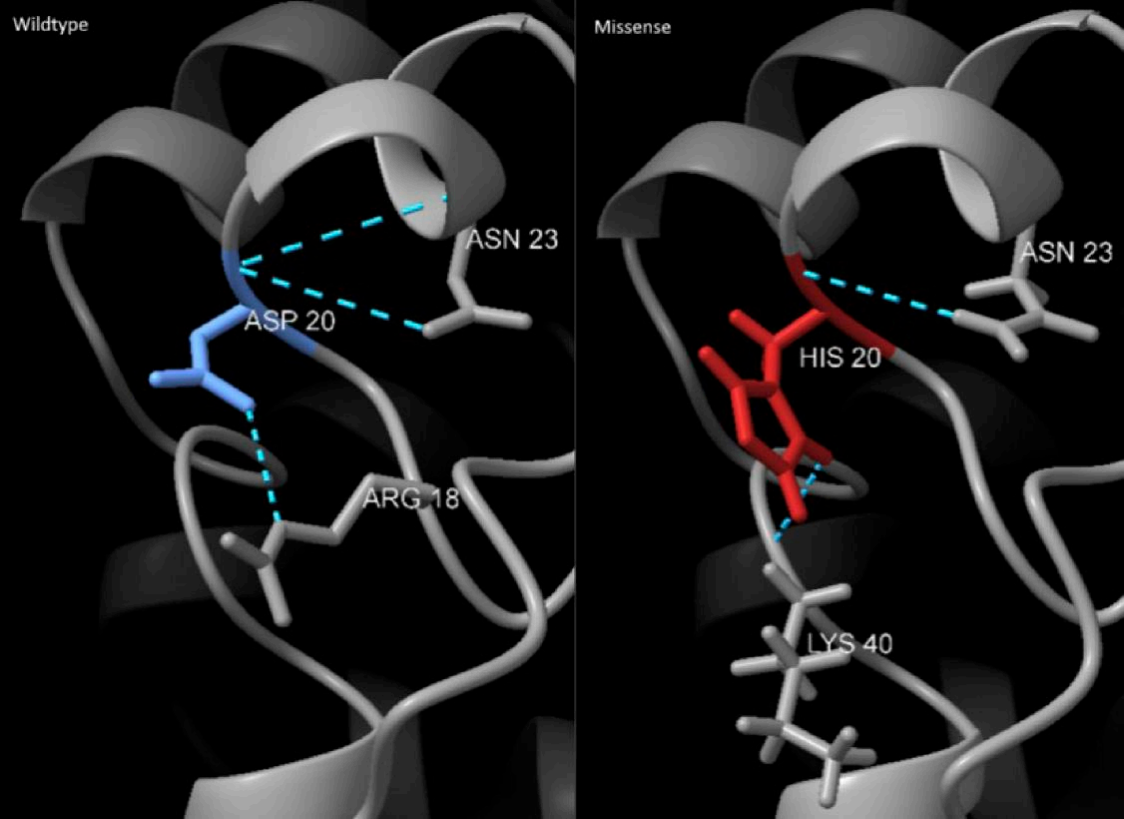
**Visual comparison of the Artemis WT and D20H mutation protein structures at the site of mutation.** The amino acid at the mutation site is color-coded, and the positions forming hydrogen bonds with it are labeled. The structures were visualized and bonds identified using UCSF ChimeraX (29, 30, 31). The wild-type structure was obtained from the AlphaFold Protein Structure Database (accession: Q96SD1) (32, 33), and the predictive 3D structure of the D20H mutant protein was generated using the AlphaFold Colab Notebook (34, 35).

Genetic analysis revealed compound heterozygous variants in *DCLRE1C* in both siblings. The paternally inherited variant was a deletion of exons 1–3, resulting in a null mutation. Deletion of exons 1–3 is one of the most common variants reported in patients with Artemis SCID (28). The maternal variant was a missense mutation causing an aspartic acid to histidine substitution in the N-terminal metallo-lactamase domain. This variant occurs in 2 out of 100,000 individuals in the European population (gnomAD) and has only been reported once previously in a patient with SCID (27). Thus, the patients in question are hemizygous for *DCLRE1C*, with the protein expressed only from the allele carrying the missense variant. This substitution is predicted to cause a structural change in the protein (Fig. S1).

Extended immunophenotyping analysis in both siblings (Table 1) showed low T cell counts, absence of B cells, few or no naive T cells, a high proportion of effector memory T cells, and normal NK cell counts. Both siblings also had a weak response to several antigens (Table 2). TCR Vβ repertoire analysis revealed an abnormal distribution with underrepresentation of several Vβ families, and the patients also lacked Vα7.2^+^ T cells (data not shown).

**Table 1.** Molecular characterization of two siblings with *DCLRE1C* variants

​	​	​		
Age at diagnosis–years	28	25		
Sex	Sister	Brother		
Clinical diagnosis	CID–leaky SCID			
Genetic variant	Compound heterozygous: Gene: *DCLRE1C* (Artemis) Refseq: NM_001033855.3 Variant 1 (maternal): Missense variant. c.58 G>C, p.Asp20His Variant 2 (paternal): NC_000010.10:g(?14984299)(15062294?)del Deletion of around 80 kb, which includes exons 1–3 in DCLRE1C ACMG classification: 5			
Malignancy	Cervical carcinoma in situ	Aggressive B cell lymphoma		

Immunological profile	Sister	Brother	Unit	Ref. interval
CD3^+^ (T cells)	**0.52** ^*^	**0.11** ^*^	×10^9^/L	0.65–1.57
CD19^+^ (B cells)	**0.01** ^*^	**<0.01** ^*^	×10^9^/L	0.08–0.28
*CD16^+^/56^+^ CD3^−^*(NK cells)	0.17	**0.62** ^*^	×10^9^/L	0.1–0.35
CD3^+^CD4^+^ (Th cells)	0.46^*^	**0.07** ^*^	×10^9^/L	0.49–1.34
CD3^+^CD4^+^ (Tc cells)	**0.06** ^*^	**0.02** ^*^	×10^9^/L	0.19–0.8
CD4^+^/CD8^+^ ratio	**7.58** ^*^	3.31	​	1.13–3.93
**Regulatory T lymphocytes**	​	​	​	​
CD4^+^CD25^+^CD127^−^	**2** ^*^	​	%	5–11
**Extended T cell panel—reference range based on healthy blood donors aged 18–65 years**	​	​	​	​
**Subtypes of CD4** ^**+**^ ** T cells**	​	​	​	​
CD4^+^/CD3^+^	**85** ^*^	67	%	44–79
CD4^+^naive (CD45RA^+^CCR7^+^)	**1** ^*^	**<0.5** ^*^	%	22–62
CD4^+^EM (CD45RA^−^CCR7^−^)	**8** ^*^	**56** ^*^	%	10–47
CD4^+^CM (CD45RA^−^CCR7^+^)	**91** ^*^	43	%	14–48
CD4^+^EMRA (CD45RA^+^CCR7^−^)	**<0.5** ^*^	**<0.5** ^*^	%	1–9
Th1 (CXCR3^+^CCR6^−^)/CD4^+^EM	26	25	%	22–52
Th2 (CXCR3^−^CCR6^−^)/CD4^+^EM	7	6	%	4–27
Th17 (CXCR3^−^CCR6^+^)/CD4^+^EM	16	**38** ^*^	%	8–28
Th1 (CXCR3^+^CCR6^−^)/CD4^+^CM	**5** ^*^	13	%	13–30
Th2 (CXCR3^−^CCR6^−^)/CD4^+^CM	**2** ^*^	**2** ^*^	%	14–53
Th17 (CXCR3^−^CCR6^+^)/CD4^+^CM	21	**43** ^*^	%	20–37
**Subtypes of CD8** ^**+**^ ** T cells**	​	​	​	​
CD8^+^/CD3^+^	**11** ^*^	**16** ^*^	%	17–47
CD8^+^ naive (CD45RA^+^CCR7^+^)	**2** ^*^	Not measurable	%	10–65
CD8^+^ EM (CD45RA^−^CCR7^−^)	**49** ^*^	Not measurable	%	4–30
CD8^+^ CM (CD45RA^−^CCR7^+^)	**47** ^*^	Not measurable	%	1–12
CD8^+^ EMRA (CD45RA^+^CCR7^−^)	**2** ^*^	Not measurable	%	18–68
**Activated T cells**	​	​	​	​
CD4^+^HLA-DR^+^CD38^−^	4	7	%	2–11
CD4^+^HLA-DR^+^CD38^+^	2	**23** ^*^	%	≤2
CD8^+^HLA-DR^+^CD38^−^	3	Not measurable	%	2–22
CD8^+^ HLA-DR^+^CD38^+^	3	Not measurable	%	1–5
**B lymphocyte subpopulations: Too few B lymphocytes to perform the analysis**	​	​	​	​

CD, cluster of differentiation; EM, effector memory; CM, central memory; EMRA, effector memory expressing CD45RA; Th, T helper cell; Tc, cytotoxic T cell; IgD, immunoglobulin D; IgM, immunoglobulin M.

* Indicates values outside the reference interval (also indicated in bold).

**Table 2.** Antigen response measured in whole blood using FASCIA

FASCIA-Lymphocyte proliferation	Sister	Brother	Unit	Ref. interval
CD4 Pokeweed mitogen	2,112	**578** ^*^	c/μl	780–3,504
CD8 Pokeweed mitogen	**18** ^*^	**28** ^*^	c/μl	104–436
CD19 Pokeweed mitogen	39	**0** ^*^	c/μl	34–775
CD4 ConA	2,505	904	c/μl	494–2,553
CD8 ConA	217	**114** ^*^	c/μl	187–2,023
CD4 Stafh. enterotoxin	**94** ^*^	**197** ^*^	c/μl	2,500–7,195
CD8 Staf. enterotoxin	**3** ^*^	**8** ^*^	c/μl	566–3,164
CD4 Tetravac	31	16	c/μl	0–300
CD8 Tetravac	6	**28** ^*^	c/μl	0–21
CD4 Purified protein derivative	**8** ^*^	16	c/μl	13–1,087
CD8 Purified protein derivative	0	12	c/μl	0–17
CD4 *Candida*	16	**7** ^*^	c/μl	11–3,248
CD8 *Candida*	0	0	c/μl	0–43
CD4 Varicella zoster	16	10	c/μl	0–231
CD8 Varicella zoster	0	0	c/μl	0–13
CD4 CMV	32	**6** ^*^	c/μl	28–4,086
CD8 CMV	**1** ^*^	33	c/μl	3–638

* Indicates values outside the reference interval (also indicated in bold).

### Use of functional assays to monitor patients’ cell function after HSCT

Both patients underwent allogeneic HSCT ∼1 year after diagnosis. Each received HLA-identical hematopoietic stem cells from the same sibling donor. The donor sibling did not carry either of the *DCLRE1C* variants detected in the patients. Conditioning consisted of a reduced dose chemotherapy-based protocol: alemtuzumab (anti-CD52) from days −10 to −3 and fludarabine from days −7 to −2, along with graft-versus-host disease (GvHD) prophylaxis with cyclosporin and mycophenolate. None of the siblings developed GvHD. The radiosensitivity of the patients’ T cells was re-evaluated using the CDA and compared to pretransplant CDA data (Table 3). T cells from both parents and an unaffected sibling were also analyzed by CDA for radiosensitivity (Table 3). At each sampling for the CDA, blood counts and T cell proliferation in response to CD3/CD28 stimulation were also measured (Table 3).

**Table 3.** Evaluation of HSCT outcome using molecular diagnostic assays

​	​	​	​	Pretransplant		​	∼1.5 years after transplant		​
​	Mother	Father	Unaffected sibling	Sister	Brother	Ref. int.	Sister	Brother	Ref. int.
**White blood cell counts**									
**WBC**	3.93	7.51	5.26	**3.05** ^*^	**2.54** ^*^	3.5–8.8	4.21	3.98	3.5–8.8
Neutrophils	2.22	3.73	2.87	**1.57** ^*^	**1** ^*^	1.8–7.5	2.91	1.7	1.6–5.9
Lymphocytes	1.26	2.73	1.7	0.924	1.02	0.8–4.5	**0.74** ^*^	**0.86** ^*^	1.1–3.5
Monocytes	0.307	0.664	0.494	0.307	0.455	0.1–1.0	0.25	0.28	0.2–0.8
Eosinophils	0.115	0.297	0.188	0.244	0.041	0.04–0.4	0.23	0.93	<0.5
Basophils	0.026	0.084	0	0.012	0.032	0–0.1	0.02	0.01	<0.1
**CD3** ^**+**^ ** (T cells)**	​	​	​	**0.52** ^*^	**0.11** ^*^	0.65–1.57	**0.43** ^*^	**0.38** ^*^	**0.65–1.57**
**CD3** ^**+**^ **CD4** ^**+**^ ** (Th cells)**	​	​	​	**0.46** ^*^	**0.07** ^*^	0.49–1.34	**0.32** ^*^	**0.33** ^*^	0.41–1.02
**CD3** ^**+**^ **CD8** ^**+**^ ** (Tc cells)**	​	​	​	**0.06** ^*^	**0.02** ^*^*	0.19–0.8	**0.1** ^*^	**0.03** ^*^	0.17–0.8
**CD4^+^/CD8^+^ ratio**	​	​	​	**7.58** ^*^	3.31	1.13–3.93	3.23	**9.83** ^*^	0.79–3.63
**T cell proliferation (%)**	90 ± 2	92 ± 10	91 ± 0	87 ± 2	**20 ± 3** ^*^	78–94 (*n* = 300)	84 ± 1	84 ± 1	78–94 (*n* = 300)
**IR sensitivity (CDA index %)**									
Dose 1 (low)^#^	67 ± 2	74 ± 1	72 ± 4	**42 ± 10** ^*^	**21 ± 4** ^*^	49–95 (*n* = 28)	61 ± 6	56 ± 7	50–97 (*n* = 40)
Dose 2 (high)^#^	13 ± 0	18 ± 2	24 ± 4	**3 ± 0** ^*^	**4 ± 0.3** ^*^	11–41 (*n* = 28)	**12 ± 1** ^*^	**11 ± 1** ^*^	15–47 (*n* = 40)
**DSB repair (%)**									
NHEJ	​	​	​	​	​	​	19.8 ± 0.3	20.1 ± 0.3	7.4–36 (*n* = 12)
HR	​	​	​	​	​	​	1.9 ± 0.1	2.1 ± 0.1	0.4–9 (*n* = 11)
**Chimerism (% patient)**									
CD3^+^	​	​	​	​	​	​	3.40%	3.60%	​
CD19^+^	​	​	​	​	​	​	3.20%	0.40%	​
CD33^+^	​	​	​	​	​	​	85.80%	80.80%	​

Reference interval for the T cell proliferation, CDA, and DNA repair assay is based on the 2.5th and 97.5th percentile of controls, and “*n*” indicates the number of controls. WBC, white blood cells.

* Indicates values outside the reference interval (also indicated in bold).

^#^ Estimated IR doses used were 1.0 Gy (dose 1) and 3.5 Gy (dose 2) for both parents, siblings, and patients prior to patient transplantation; for posttransplant samples, IR doses were 0.36 Gy (dose 1) and 1.95 Gy (dose 2).

The parents had normal blood counts, normal T cell proliferation indices, and no significant radiation sensitivity, indicating that each variant alone does not confer radiosensitivity.

Before HSCT, both patients had slightly low white blood cell counts due to low neutrophil levels. After HSCT, total white blood cell counts were within the normal range, but total lymphocyte counts were somewhat lower than the reference interval. The immunophenotype analyses of T cell populations, based only on CD4 and CD8 markers, indicated cell counts lower than the reference interval for all cell populations, even after the HSCT (Table 3), but extended T cell immunophenotying was not carried out after HSCT.

**Figure S2.**
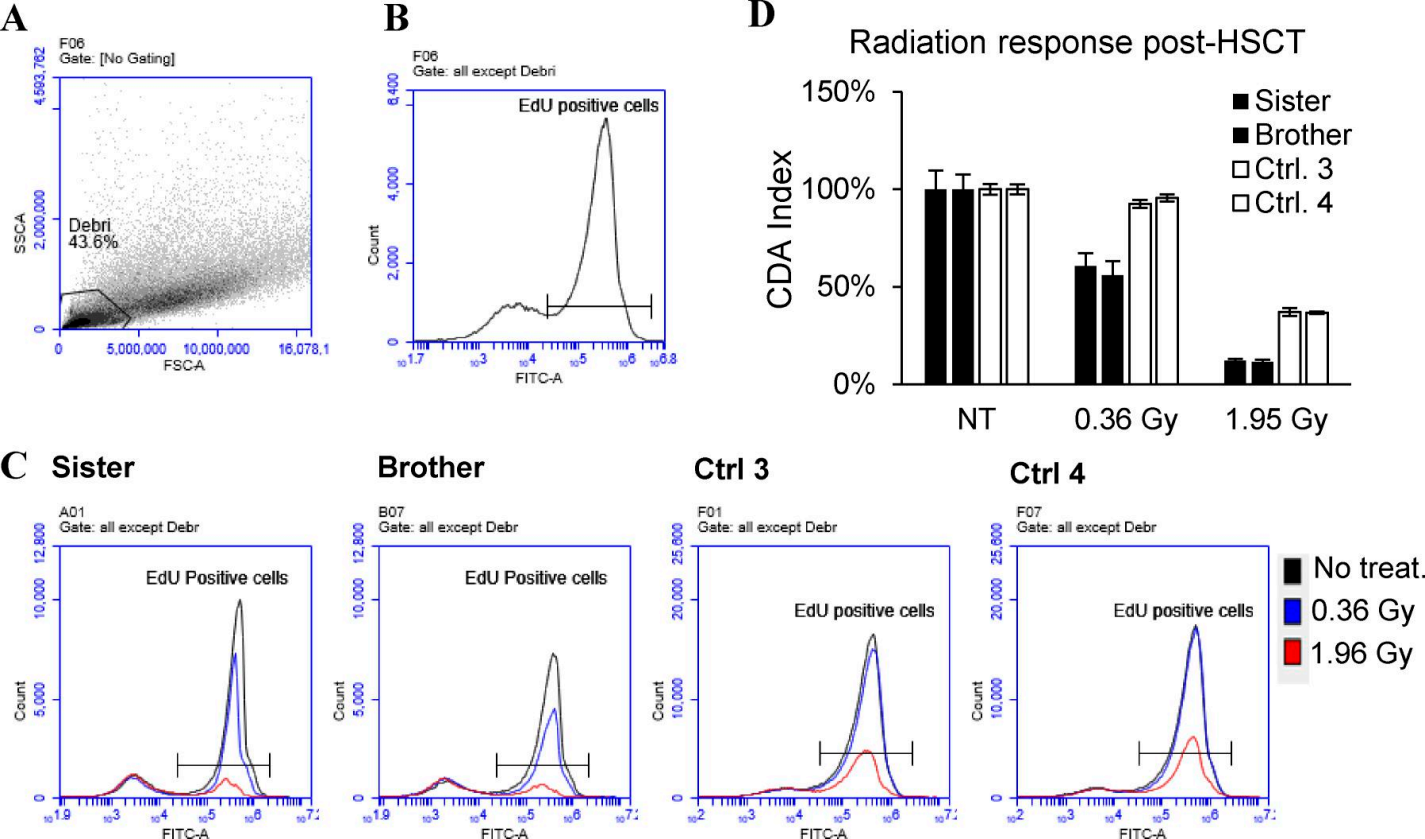
**Gating strategy and representative flow cytometry plots for the CDA. (A)** Debris was excluded based on forward-side scatter plots. **(B)** Histogram of events versus fluorescence in the FITC channel was used to distinguish the EdU-Alexa Fluor 488–positive cells. **(C)** Representative histograms for the two siblings with variants in DCLRE1C and two heathy controls treated with the indicated doses of radiation or left untreated after HSCT. **(D)** Bar graph of the CDA index (no. of EdU-positive cells in treated sample/no. of EdU-positive cells in untreated sample [%]) for the two siblings after HSCT and two heathy controls treated with radiation at the indicated doses. The controls were assigned arbitrary numbers. Error bars indicate the standard deviation of three technical replicates for the patients and two replicates for the controls.

Before HSCT, T cell proliferation was significantly low in the brother’s sample but normal for the sister when compared to the reference interval for the assay (Table 3). After HSCT, T cell proliferation was normal in both. The in vitro sensitivity to radiation was investigated via CDA at two doses. The T cells from both siblings were sensitive at both doses prior to HSCT (Table 3). After HSCT, T cells from both patients were still RS, but only at the higher dose of the radiation, and the radiosensitivity was less than the measurements prior to HSCT, as judged relative to the reference interval (Table 3 and Fig. S2 D).

**Figure S3.**
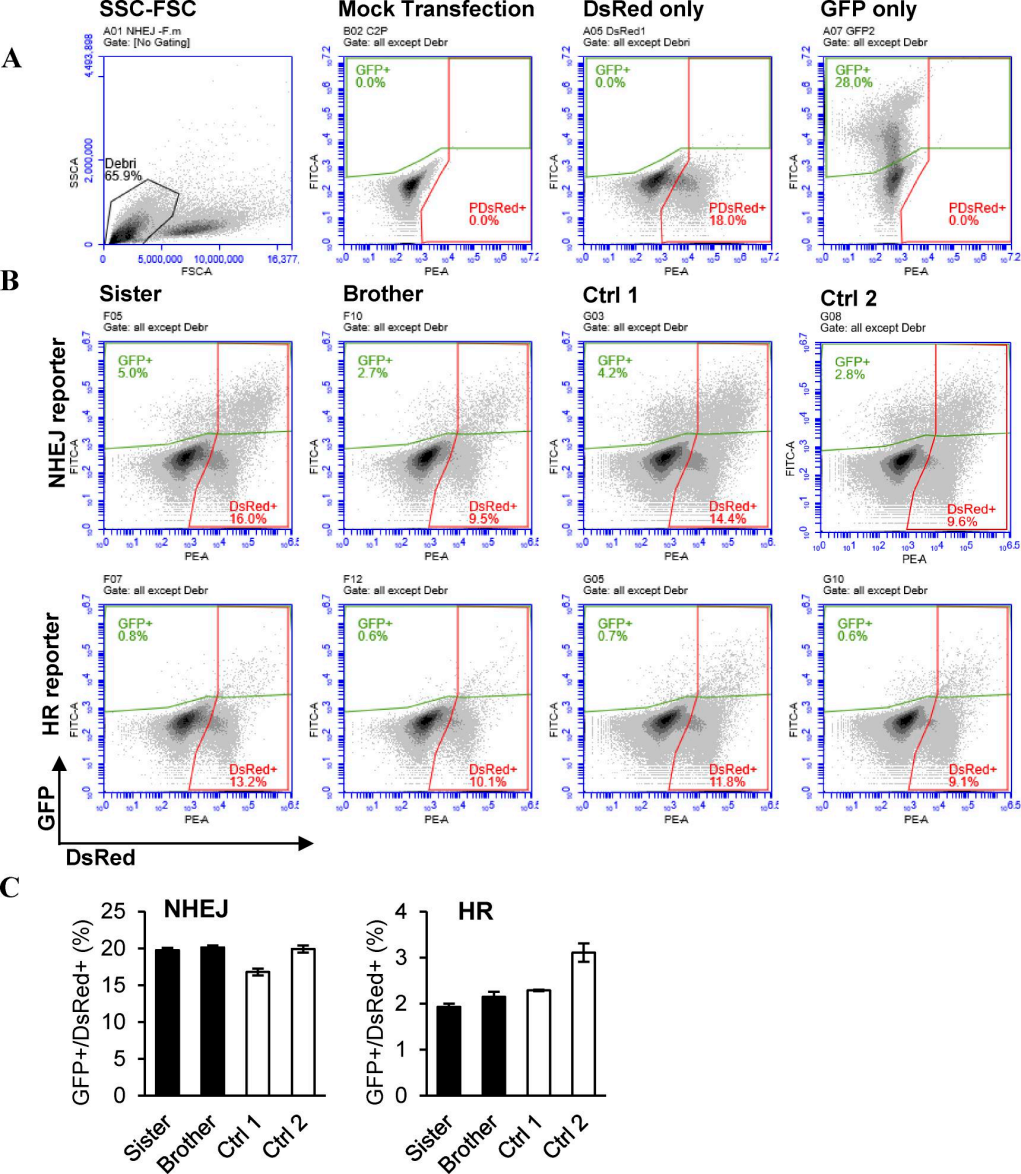
**Gating strategy and representative flow cytometry plots for the NHEJ and HR measurements. (A)** Debris was excluded based on forward-side scatter plots (left panel). Single transfections of mock transfected, DsRed alone, or GFP alone were used to determine the positive populations in each channel. **(B)** Representative histograms for the two siblings with variants in DCLRE1C and two healthy controls transfected with either NHEJ or HR reporter plasmid. **(C)** Bar graph of the repair efficiency (no. of GFP-positive cells/no. of DsRed-positive cells [%]) for the two siblings after HSCT and two healthy controls. The controls were assigned arbitrary numbers. Error bars indicate the standard deviation of two technical replicates.

To evaluate the T cells’ capacity to repair DSBs, an in-house assay modified from a previously published protocol was used (26). The level of HR and NHEJ repair pathways in patient cells was comparable to controls after HSCT (Table 3 and Fig. S3 C).

**Figure S4.**
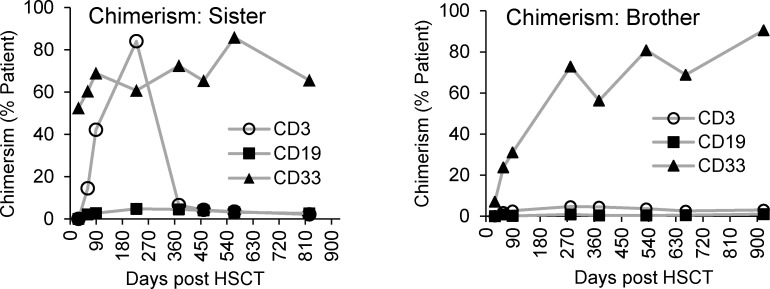
**Chimerism in enriched blood cell populations, CD3** ^**+**^ **, CD19** ^**+**^ **, and CD33** ^**+**^ **cells were determined using NGS at different time points** **after** **HSCT.** The values indicate the % of patient cells still present.

Chimerism was measured at multiple time points after HSCT (Table 2 and Fig. S4). The data indicated low myeloid engraftment based on high levels of patient-derived CD33^+^ cells, while T cells (CD3^+^) and B cells (CD19^+^) were mostly of donor origin.

## Discussion

Patients with disease-causing variants in both alleles of *DCLRE1C* often present with severe SCID and clinical manifestations during early infancy. Hypomorphic variants, which preserve some function of the altered protein, may lead to diagnosis later in life with presentations such as Omenn syndrome, hyper-IgM syndrome, or CID with milder or atypical features, known as leaky SCID. Given the late age of clinical onset for the patients investigated here, who harbor a null variant along with a missense variant, it is likely that the missense variant results in a protein with residual activity, although in vitro Artemis activity was not investigated. Thus, the clinical and molecular manifestation of the variants in *DCLRE1C* in the siblings presented here, with an immunophenotype corresponding to a defective V(D)J recombination and cellular radiosensitivity, supports a diagnosis of CID/leaky SCID (22).

The missense variant in *DCLRE1C* was predicted to affect the structure of the protein is in the N-terminal metallo-lactamase domain, which is required for its nuclease activity. This nuclease activity is essential for the processing of DNA ends in the repair of DSBs via the NHEJ pathway as well as for resolving DSB intermediates generated by RAG1 and RAG2 during early V(D)J recombination (36, 37). The observed hypersensitivity of patient cells to radiation treatment prior to HSCT suggests a defect in the nuclease activity of the missense variant.

Standard conditioning with DNA-damaging alkylating agents before HSCT increases the risk of long-term toxicity in patients with DSB repair defects, such as Artemis deficiency (20). Conversely, patients with SCID due to Artemis deficiency who receive unconditioned HSCT often fail to reconstitute B cells and require life-long immunoglobulin replacement therapy (38, 39). Patients with SCID who receive HSCT later in life have been reported to have poorer outcomes (40, 41). The siblings described here were conditioned with a reduced-intensity chemotherapy protocol. At ∼1.5 years after transplant, both had high levels of patient-derived myeloid chimerism and low peripheral lymphocyte counts, but T cell engraftment exceeded 95%. We investigated the functional characteristics of patient T cells after HSCT as a complement to chimerism analyses. After transplant, T cell proliferation was normal in both patients, indicating a favorable outcome, especially for the brother, who had very low cell proliferation before transplantation. Surprisingly, T cells from both patients remained RS, although this was detectable only at the higher radiation dose, and sensitivity levels were less pronounced than before HSCT. This residual radiosensitivity may be due to the residual patient T cells present, although CD3^+^ cell chimerism was below 5% in both patients. The CDA is designed to support specific T cell activation and proliferation, so the contribution from other cell populations is unlikely, although not experimentally ruled out. The apparent radiosensitivity of T cells only at the higher dose is of unclear clinical significance, and further studies of patients with HSCT will be required to address whether this is donor specific or common to patients with HSCT. Nonetheless, the CDA indicated the ability of the T cells from the donor to proliferate normally and showed an improvement in T cell radiation sensitivity posttransplant, complementing the chimerism analyses.

Artemis recognizes single- to double-strand DNA transitions as targets for its endonucleolytic activity (42). We measured DSB repair efficiency using transfected reporter plasmids linearized to mimic this DNA structure in donor-derived T cells. The results showed similar repair efficiency in patient and control T cells, suggesting that radiosensitivity in donor T cells is not due to a pronounced NHEJ defect. Unfortunately, the assay was not available prior to the transplantation, which would have provided a more relevant reference for the assay. Overall, the patients described here have shown a satisfactory clinical outcome and no longer require immunoglobulin replacement therapy, indicating sufficient immune reconstitution at the time of evaluation. This study indicates that the CDA and DSB repair efficiency measurements correlate with the reconstitution of T cells and immune response in patients after HSCT, providing proof of concept for their application and complementing the chimerism analyses. Furthermore, in cases of autologous HSCT transplantation of genetically corrected autologous cells, chimerism cannot be used to detect engraftment. Thus, these assays may become an important tool in the evaluation of the T cell function after transplantation in these cases.

Reporting the functional and clinical pathogenesis of novel SCID variants before and after HSCT is critical for future diagnoses and individualized treatment strategies. The novel functional assays reported here were developed for clinical use and are likely to become valuable diagnostic tools.

## Materials and methods

### Data obtained from patients’ medical records

#### Genetic analyses

Whole-genome sequencing was performed for the sister at the Science for Life Laboratory in Solna. The data were analyzed using software from the reference laboratory (SCOUT, Clinical Genomics, Scilife Solna). The analysis was limited to known monogenic immunodeficiency–associated genes, examining variants in exons and exon–intron boundaries. The identified disease-causing variants were then verified by targeted genetic analysis using Sanger sequencing and multiplex ligation-dependent probe Amplification to assess sequence variants and copy number changes for both siblings.

**Figure S5.**
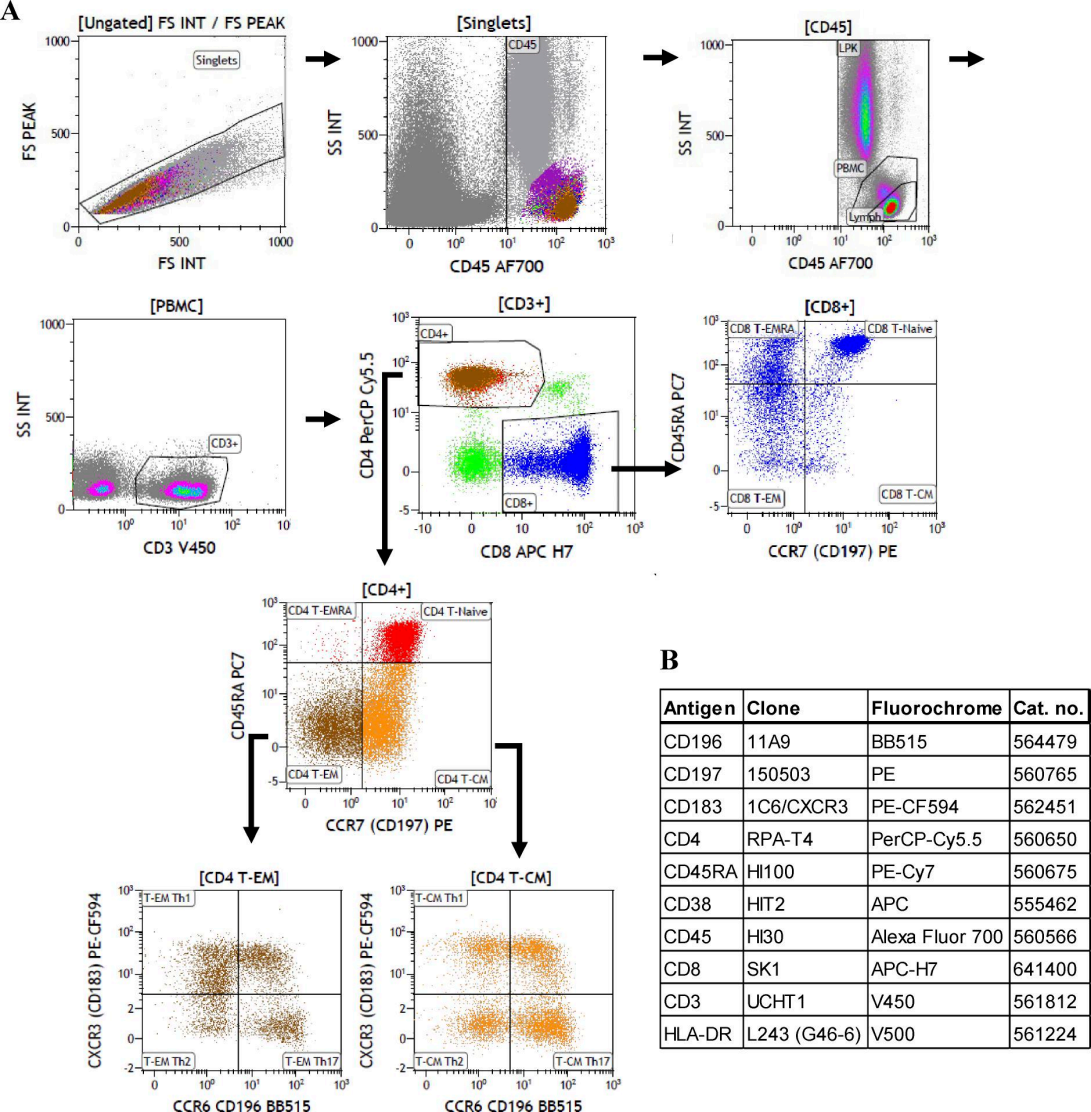
**Extended immunophenotyping of T cells. (A)** Representative gating strategy for the extended T cell panel obtained from Clinical Immunology, Medical Diagnostics Center, Karolinska University Hospital. **(B)** List of antibodies used in the panel. The antibodies were purchased from BD Biosciences.

Immunophenotyping was carried out by Clinical Immunology, Medical Diagnostics Center, Karolinska University Hospital. Extended flow cytometric immunophenotyping was performed for both patients. The analysis included in-depth characterization of lymphocyte subsets, including T cell differentiation and activation profiles, regulatory T cells, B cell maturation stages, and overall immune cell composition. TCR Vβ repertoire analysis was also performed to evaluate T cell diversity. The analyses were conducted according to established clinical protocols and reference standards on Beckman Coulters: AQUIOS, CytoFLEX, and Navios machines. A representative gating strategy for the extended T cell panel and the antibodies used are presented in Fig. S5.

Antigen response analysis was carried out using FASCIA by Clinical Immunology, Medical Diagnostics Center, Karolinska University Hospital. FASCIA is a flow cytometry–based method for evaluating cell-mediated immune response in whole blood.

Chimerism analyses were carried out by Clinical Immunology, Medical Diagnostics Center, Karolinska University Hospital. NGS was used to identify genetic differences between donor and recipient for chimerism assessments. The analysis was performed on enriched blood cell populations, including CD3^+^ T cells, CD19^+^ B cells, and CD33^+^ myeloid cells. The purity of isolated cell populations was not routinely verified. All procedures were carried out according to the laboratory’s standard protocols.

### Blood sampling for CDA and DSB repair efficiency measurements

EDTA blood samples from patients, parents, and an unaffected sibling were collected. All blood samples were processed within 8 h.

Excess blood (EDTA tubes) with normal blood counts from routine hematology at the Sahlgrenska University Hospital central laboratory were used as controls. The controls were randomly selected, included both sexes, and their ages ranged from 18 to 75 years.

### CDA

CDA was carried out as previously described (23). Briefly, peripheral blood mononuclear cells (PBMCs) were isolated from whole blood using Lymphoprep (Catalog #07811; STEMCELL Technologies) according to the manufacturer’s instructions. In vitro irradiation of PBMCs was performed using an RS 3400 Rad Source X-ray Blood Irradiator (Rad Source Technologies), with lead shielding of varying thicknesses estimated to result in two doses: 0.36 and 1.95 Gy. Notably, IR treatment doses carried out prior to the HSCT were estimated to be 1.0 and 3.5 Gy, resulting in a difference in the reference intervals at these two time points. Treatments were performed in triplicate for patient cells and in duplicate for controls. Cells were seeded in 24-well plates at 250 × 10^3^ cells in 500 μl per well. T cells were stimulated with Human T-Activator CD3/CD28 Dynabeads (Cat# 11132D) and cultured in GIBCO OpTmizer CTS T-Cell Expansion SFM (Cat# A10485-01), supplemented with 0.4 nM IL-2 (Cat# PHC0026) for 72 h (Thermo Fisher Scientific), following the manufacturer’s instructions. After 72 h, 500 μl of fresh media and 10 µM EdU (5-ethynyl-2′-deoxyuridine) (Cat# A10044; Thermo Fisher Scientific) were added, and cells were incubated for another 16–18 h. Cells were fixed by adding 1% formaldehyde (Cat# F8775-500ML; Merck) for 15 min at room temperature, washed with PBS containing 0.1% bovine serum albumin (PBS-BSA) buffer, and permeabilized using saponin permeabilization and wash buffer (SPWB) (0.1% saponin in PBS-BSA). After 30 min, cell pellets were resuspended in a Click-iT reaction mix, prepared immediately before use: 2 mM CuSO_4_ (Merck Millipore), 0.4 µM Alexa Fluor 488 azide dye (Cat# A10266; Thermo Fisher Scientific), and 10 mM sodium ascorbate (Merck) in PBS. The cells were incubated for 30 min.

Cells were then resuspended in 200 μl SPWB supplemented with Vybrant DyeCycle Ruby (1:1,000) (Cat# V10273; Thermo Fisher Scientific) and RNase A (0.5 mg/ml) (Cat# 19101; Qiagen) and incubated for 20 min. CD3/CD28 beads were removed using a magnetic concentrator. Samples were analyzed using an Accuri C6 Plus cytometer (BD Biosciences) in fast mode (optical filters: 533/30 and 675/25). Data were analyzed using BD Accuri C6 software, as described previously (23). The T cell proliferation rate was calculated as the percentage of EdU-positive cells in the untreated sample after the 16-h incubation. Flow cytometry files from pre- and post-HSCT analyses were reanalyzed together, and reference intervals were defined as described in the data analysis section. A representative gating strategy for the CDA analyses is presented in Fig. S2.

### DSB repair efficiency measurements

Plasmids were kindly provided by Professor Vera Gorbunova, University of Rochester, Rochester, NY, USA. Reporter plasmids for NHEJ and HR pathways were linearized using I-SceI (Cat# R0694S; BioNordika) and purified using the QIAEX II gel extraction kit (Qiagen), as described by Seluanov et al*.* (26). T cells cultured for 72 h as described in the CDA were used for transfection. 1 × 10^6^ cells were transfected with 2.5 µg of linearized reporter DNA and 0.5 µg of DsRed plasmid in a total volume of 100 μl, using a 4D-Nucleofector system (Lonza Bioscience). The P3 Primary Cell 4D-Nucleofector kit (Cat# V4XP-3024; BioNordika) was used with program EO-115 for electroporation. Cells were subsequently cultured for an additional 48 h.

Cells were harvested, resuspended in PBS-BSA, and analyzed on a BD Accuri C6 Plus flow cytometer using fast settings (optical filters 510/15 for GFP and 585/40 for DsRed). Fluorescence spillover was corrected using a compensation matrix, calculated using cells transfected with either pMax-GFP (Plasmid #177825) or DsRed alone. The compensation values applied are listed in Table 4.

**Table 4.** Compensation values applied

Detector	Compensated from	Compensation value
FL1 (GFP)	FL2 (DsRed)	2.5%
FL2 (DsRed)	FL1 (GFP)	20%

A representative gating strategy for the DNA repair efficiency analysis is presented in Fig. S3.

### Data analysis

The data were analyzed using GraphPad Prism 10.4.1 version for Windows (GraphPad Software, https://www.graphpad.com/). Mean and standard deviation from at least two replicates were used for all individuals analyzed by the CDA, T cell proliferation, and DNA repair assays. Reference intervals were based on the 2.5th and 97.5th percentiles of control samples. Individual values outside this 95% interval were considered significant.

### Use of generative AI and AI-assisted technologies in the writing process

During the preparation of this work, the author(s) used OpenAI/ChatGPT (June 2024) to specifically correct grammatical errors. After using this tool/service, the author(s) reviewed and edited the content as necessary and take full responsibility for the content of the publication.

### Online supplemental material

Fig. S1 provides a visual comparison of the predicted Artemis WT and D20H mutation protein structures at the site of mutation. Fig. S2 provides the CDA gating strategy, representative flow cytometry plots, and a bar graph of radiation response in samples from the two siblings after HSCT and two healthy controls. Fig. S3 shows the gating strategy and representative flow cytometry plots for the NHEJ and HR measurements, as well as bar graphs of the repair efficiency for the two siblings after HSCT and two healthy controls. Fig. S4 is a graph of chimerism in enriched blood cell populations, CD3^+^, CD19^+^, and CD33^+^, measured by NGS at several time points after HSCT. Fig. S5 provides a representative gating strategy for the extended T cell panel obtained from routine clinical immunology at Karolinska University Hospital.

## Ethical statement

The study was approved by the Regional Ethical Review Board in Gothenburg and the Swedish Ethical Review Authority.

## Consent for publication

Written informed consent was obtained from the patients and family members.

## Data Availability

The datasets generated during and/or analyzed during the current study are available from the corresponding author upon reasonable request.
